# Gallstone ileus: an unusual cause of mechanical intestinal obstruction in an elderly woman

**DOI:** 10.11604/pamj.2022.42.40.35278

**Published:** 2022-05-16

**Authors:** Tarik Souiki, Khalid Mazaz

**Affiliations:** 1 Faculty of Medicine and Pharmacy, Sidi Mohammed Ben Abdellah University, Fez, Morocco,; 2 Department of Visceral Surgery, University Hospital Hassan II, Fez, Morocco

**Keywords:** Gallstone ileus, intestinal obstruction, enterotomy

## Image in medicine

Gallstone ileus is a rare cause of intestinal obstruction. It is due to biliary-enteric fistula complicating cholelithiasis. It occurs mostly in elderly women with a heavy history of associated comorbidities. The clinical presentation is characterized by insidious and intermittent obstructive symptoms, which often might delay diagnosis. The mainstay treatment is surgical, which consisted of an urgent enterolithotomy allowing prompt relief of obstruction. The management of bilio-enteric fistula is not mandatory in the same procedure and is discussed based upon patient characteristics. Herein, we report a female case of 80-year-old, with a history of diabetes mellitus and morbid obesity. The patient presented with an abdominal acute pain associated with vomiting evolving along five days. The physical examination showed a heart rate of 110 beats per minute, blood pressure of 110/70 mmHg and body temperature of 37.6°C. The abdominal examination revealed a slightly tympanic abdomen with diffuse tenderness. The initial biological blood assessment showed a slight increase of C-reactive protein at 21 mg/L, and leukocytosis of 14x10^9^ elements/L. Other blood investigations were normal. The CT scan of the abdomen showed dilated small bowel loops upstream of a large gallstone with a calcified rim. These findings are consistent with gallstone ileus (A). After adequate resuscitation with intravenous fluids, the patient underwent an urgent midline mini-laparotomy. The surgical exploration revealed the presence of a gallstone in the ileus resulting in an intestinal obstruction (B). The gallstone was removed through longitudinal enterotomy and the defect was closed transversally (C). Given the high anesthetic risk for the patient and the need for a shortened laparotomy, it has been decided not to treat the cholecystitis and the enterobiliary fistula. The patient had an uneventful recovery and has no symptoms within 6 months follow-up.

**Figure 1:**
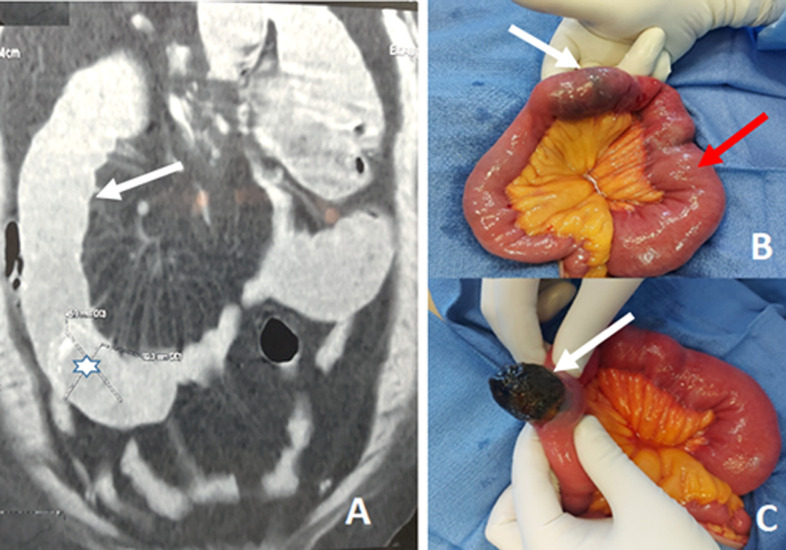
A) abdominal computed tomography scan (coronal section), showing dilated loops (arrow) of small bowel with a large gallstone with a calcified rim (star); B) per-operative view showing gallstone impacted in the ileus (white arrow) with dilated intestinal proximal loops (red arrow); C) per-operative view showing extraction of the gallstone through a longitudinal enterotomy (white arrow)

